# The diet-microbiota-inflammation axis and colorectal cancer

**DOI:** 10.3389/fonc.2026.1895753

**Published:** 2026-07-15

**Authors:** Konstantinos Kossenas, Christos Damaskos, Nikolaos Garmpis

**Affiliations:** 1 Laiko General Hospital, Athens, Greece; 2 Army Share Fund Hospital (NIMTS)417 Military Hospital, Athens, Greece; 3 Colorectal Department Guy’s and St. Thomas Hospital, London, United Kingdom

**Keywords:** colon, diet, *Fusiobacterium nucleatum*, inflammation, mechanisms, rectal, western

## Abstract

**Background:**

Colorectal cancer (CRC) is still one of the leading causes of cancer morbidity and mortality worldwide. There is increasing evidence that diet, gut microbiota, microbial metabolites and chronic inflammation are important factors in colorectal carcinogenesis and may provide novel opportunities for prevention, diagnosis and treatment.

**Aim:**

To provide a comprehensive review of the current evidence on the role of diet, nutrition, microbial metabolism and chronic inflammation in CRC, with emphasis on emerging translational applications including microbiome-based biomarkers and microbiota-targeted therapeutic strategies.

**Methods:**

A literature search was performed with PubMed, Scopus and the Cochrane Library. Relevant studies on diet-microbiota interactions, microbial metabolites, inflammatory mechanisms, colorectal carcinogenesis, microbiome-derived biomarkers, and microbiota-targeted interventions were identified and reviewed. Preclinical and clinical studies and high quality reviews and meta-analyses were considered.

**Results:**

Dietary patterns have been shown to have a major impact on the composition and function of the gut microbiota. Rich-fiber diets and short-chain fatty acids (SCFAs) production seem protective against CRC, while western dietary patterns, ultra-processed foods and dysbiosis-associated metabolites promote a pro-inflammatory environment associated with carcinogenesis. Some microorganisms such as *Fusobacterium nucleatum*, enterotoxigenic Bacteroides fragilis and pks-positive Escherichia coli have been associated with CRC by inflammatory, genotoxic and immune-modulatory mechanisms. Recent advances in sequencing technologies and multi-omics approaches have enabled the identification of microbial signatures with potential diagnostic and prognostic value. Moreover, microbiota-targeted interventions such as probiotics, prebiotics, postbiotics, faecal microbiota transplantation, and next-generation microbial therapies have yielded promising preclinical and early clinical results.

**Conclusions:**

The diet-microbiota-inflammation axis is a key player in colorectal carcinogenesis and a promising target for translational research. Microbiome-based biomarkers and microbiota-targeted therapies may have a role in future precision prevention and personalised management strategies of colorectal cancer despite significant challenges in terms of causation, standardisation and translation into clinical practice.

## Introduction

Colorectal cancer (CRC) is still one of the most frequently diagnosed malignancies worldwide and is a major cause of cancer-related morbidity and mortality (1). Even with great strides in screening programs, surgical techniques, systemic therapies, and preventive strategies, CRC remains a significant global health burden (1, 2). Moreover, the rising incidence of CRC in younger subjects has raised a considerable concern and emphasises the need for a better understanding of the environmental, lifestyle and biological factors involved in colorectal carcinogenesis (3).

CRC has been traditionally considered as a disease caused by the accumulation of genetic and epigenetic changes in key molecular pathways that control cellular proliferation, differentiation and survival (4). However, there is growing evidence that environmental and lifestyle factors may also play important roles in the development and progression of CRC (5). Among these factors, diet has been recognised as one of the most important modifiable determinants of CRC risk. High intake of red and processed meat and ultra-processed foods has been linked to an increased risk of CRC, whereas dietary patterns rich in fibre, fruits, vegetables and whole grains appear to have protective effects (6–8).

More recently, the gut microbiota has garnered greater attention as a potential mediator connecting dietary exposures, host metabolism, immune responses and colorectal carcinogenesis (9). The human gastrointestinal tract harbours a complex microbial community which participates in nutrient metabolism, the maintenance of epithelial barrier integrity and the regulation of mucosal immunity (10). Dysbiosis, i.e. alterations in the composition and function of these microbial communities, has been consistently associated with CRC (11). Furthermore, specific microbial taxa and microbial-derived metabolites have been implicated in processes related to tumour initiation, progression and modulation of host inflammatory responses (10, 12–14).

There is a growing appreciation that chronic inflammation may provide a mechanistic link between dietary exposures, microbial dysbiosis and colorectal carcinogenesis. Persistent inflammatory signalling can induce epithelial damage, oxidative stress, immune dysregulation and genomic instability, which provides a microenvironment favouring malignant transformation and tumour progression (14, 15). Furthermore, microbial metabolites have been shown to be important mediators of host-microbiota interactions and to be involved in immune responses, cellular signalling pathways, and cancer-related processes (16, 17). There is existing evidence that multiple stages of colorectal cancer development are the result of complex interactions of dietary factors, microbial communities, microbial metabolites, and host immune pathways (18–22).

Great interest has been generated for the translational potential of the diet-microbiota-inflammation axis, with increasing appreciation for the axis. The recent advances in metagenomics, metabolomics and systems biology have allowed the identification of microbial signatures associated with CRC and increased the opportunities for the development of new biomarkers and microbiome-targeted interventions (23, 24). Hence, the aim of this narrative review is to summarise current evidence on the role of diet, nutrition, microbial metabolism, and chronic inflammation in colorectal cancer and to highlight emerging translational applications, including microbiome-based biomarkers and microbiota-targeted therapeutic strategies.

## Methods

### Literature search strategy

The purpose of this narrative review was to summarise current evidence regarding the role of diet, nutrition, microbial metabolism, gut microbiota and chronic inflammation in colorectal carcinogenesis and their emerging translational applications in colorectal cancer (CRC).

We conducted a thorough literature search in PubMed, Scopus, and the Cochrane Library from inception to May 2026. Search terms were developed using a combination of Medical Subject Headings (MeSH) and free-text keywords related to colorectal cancer, gut microbiota, diet, nutrition, microbial metabolites, chronic inflammation, dysbiosis, short-chain fatty acids, biomarkers, probiotics, prebiotics, postbiotics, faecal microbiota transplantation, and precision medicine. The search strategy was optimised using the Boolean operators (“AND”, “OR”).

### Study selection

Titles and abstracts of articles were screened, and full-text evaluation was performed when appropriate. We focused on studies that directly examined the relationship between diet, microbial composition, microbial metabolites, inflammation and colorectal carcinogenesis. Studies included were experimental, observational, clinical trials, systematic reviews, meta-analyses and high-quality narrative reviews.

We excluded studies that were not directly related to colorectal cancer, the gut microbiota, dietary influences, inflammatory mechanisms, or translational microbiome applications. We also applied the following exclusion criteria: non‐English articles; conference abstracts lacking sufficient methodological detail; and studies that were not relevant to the objectives of the review.

### Data synthesis

As this review is narrative, no formal quantitative synthesis or meta-analysis was conducted. The qualitative synthesis of the evidence was organised into three major thematic domains: (1) diet, nutrition, microbial metabolism and colorectal cancer risk; (2) gut microbiota, chronic inflammation and colorectal carcinogenesis; and (3) translational applications including microbiome-based biomarkers and microbiota-targeted therapeutic interventions. Particular emphasis was placed on mechanistic studies, translational research and clinically relevant findings that may inform future precision prevention and treatment strategies in CRC.

## Results

### Diet, nutrition, microbial metabolism and risk of colorectal cancer

Dietary factors have been recognised for a long time as important modulators of CRC risk. High consumption of red and processed meat is associated with an increased risk of colorectal neoplasia, whereas diets rich in fibre, whole grains, fruits, vegetables, calcium and dairy products are associated with reduced risk (25). These results are consistent with the hypothesis that diet affects carcinogenesis via multiple mechanisms, including modulation of inflammation, obesity-related pathways, immune response, and gut microbiota interactions. Importantly, dietary exposures are rarely isolated. Rather, the overall dietary patterns seem to have cumulative effects on microbial composition and metabolic outputs that affect susceptibility to colorectal cancer.

One of the most studied links between diet and CRC is dietary fibre and the generation of short-chain fatty acids (SCFAs). Undigested complex carbohydrates arrive at the colon where they are fermented by gut microorganisms to produce SCFAs including acetate, propionate and butyrate (26). Among these metabolites, butyrate has been attracting a lot of attention because of its potent anti-inflammatory and anti-neoplastic properties. Butyrate is the main energy substrate for colonocytes, preserves the integrity of the epithelial barrier, modulates the immune response and influences gene expression through epigenetic mechanisms (26). It has been experimentally demonstrated that butyrate can suppress colorectal carcinogenesis through the inhibition of inflammation and the promotion of epithelial homeostasis.

Singh and colleagues highlighted the importance of butyrate signalling, demonstrating that butyrate and niacin activation of the GPR109A receptor suppresses colonic inflammation and carcinogenesis (27). GPR109A signalling promotes anti-inflammatory phenotypes in macrophages and dendritic cells, induces regulatory T-cell differentiation, and drives IL-18 production in the colonic epithelium. Mice lacking this receptor were more susceptible to colitis and colon cancer, highlighting the important role of microbial metabolites from dietary sources in preventing CRC development (27).

The beneficial effects of SCFAs are highly dependent on the presence of specialised SCFA-producing bacteria in the gut microbiota. Several bacterial taxa, such as Faecalibacterium, Roseburia, Eubacterium, and Anaerostipes species, are the main contributors to butyrate production (28). These microorganisms help to maintain gut homeostasis and regulate inflammatory pathways. SCFA-producing bacteria have been consistently reported to be reduced in numbers in individuals with colorectal cancer and other chronic inflammatory disorders, suggesting that the loss of these beneficial microbes may contribute to disease pathogenesis (28).

Diet is a major driver of the abundance and metabolic activity of these protective microbial communities. Fiber-rich dietary patterns promote microbial diversity and increase populations of beneficial fermentative bacteria (29). In contrast, Western dietary patterns, which are normally high in saturated fats, refined carbohydrates and processed foods, have been associated with microbial dysbiosis and decreased production of beneficial metabolites. Western dietary patterns have been associated with dysbiosis and chronic inflammatory conditions that may contribute to CRC development (30) (**Tables 1** and **2**).

**Table 1:** Dietary/microbial factors, their mechanisms and the effect on CRC risk.

Topic	Dietary/microbial factor	Mechanism	Effect on CRC risk	Sources
Protective diet	Fiber, whole grains, fruits, vegetables, calcium, dairy	Reduced inflammation, favorable microbiota, immune modulation	↓ CRC risk	(25)
Harmful diet	Red and processed meat	Pro-inflammatory and carcinogenic metabolites	↑ CRC risk	(25)
SCFAs	Acetate, propionate, butyrate	Maintain epithelial barrier, regulate immunity, epigenetic modulation	↓ CRC risk	(26)
Butyrate-GPR109A signaling	Butyrate, niacin	Treg induction, IL-18 production, anti-inflammatory signaling	↓ Colitis and carcinogenesis	(27)
SCFA-producing bacteria	Faecalibacterium, Roseburia, Eubacterium, Anaerostipes	Butyrate production and maintenance of gut homeostasis	Protective	(28)
High-fiber diet	Increased fermentable substrates	Increased microbial diversity and SCFA production	↓ CRC risk	(29)
Western diet	Saturated fat, refined carbohydrates, processed foods	Dysbiosis, reduced SCFAs, increased permeability	↑ CRC risk	(30)
Mediterranean diet	Fruits, vegetables, legumes, whole grains, olive oil, fish	Favorable gut microbial composition and anti-inflammatory effects	↓ CRC risk	(30)
Ultra-processed foods	Additives, emulsifiers, refined sugars, unhealthy fats	Dysbiosis, barrier dysfunction, chronic inflammation	↑ CRC risk	(31)
Protein fermentation	Animal protein metabolism	Production of ammonia, hydrogen sulfide, phenols, amines	↑ CRC risk	(26)
Secondary bile acids	Fat-induced bile acid metabolism	DNA damage, oxidative stress, tumor promotion	↑ CRC risk	(26)
Metabolic reprogramming	Diet-microbiota-host interactions	Alters intestinal stem-cell metabolism and tumor microenvironment	Promotes CRC initiation/progression	(32)
Prebiotics	Selective stimulation of beneficial bacteria	Increased bifidobacteria and SCFA production	Potentially protective	(33)

**Table 2:** Mechanisms, pathways and CRC development.

Factor/pathogen	Mechanism	Molecular pathways	Consequence for CRC development	Sources
Dysbiosis	Reduced microbial diversity, loss of beneficial taxa, expansion of pathogenic species	Immune dysregulation, chronic inflammation	Creates pro-tumorigenic microenvironment	(34, 35)
Driver-Passenger Model	Early pathogenic bacteria initiate carcinogenesis; later bacteria colonize tumor niche	DNA damage, inflammation, epithelial dysfunction	Tumor initiation and progression	(34)
*Fusobacterium nucleatum*	Promotes inflammation, proliferation, invasion, and immune evasion	NF-κB activation, IL-6, IL-8, TNF-α secretion	Tumor growth, invasion, immune suppression	(34, 36, 37)
Enterotoxigenic *Bacteroides fragilis* (ETBF)	Produces Bacteroides fragilis toxin (BFT)	IL-17 production, STAT3 activation	Chronic inflammation and tumor promotion	(34)
pks+ *Escherichia coli*	Produces colibactin genotoxin	DNA double-strand breaks, mutational signatures	Genomic instability and carcinogenesis	(36, 37)
Chronic Inflammation	Persistent cytokine and ROS production	NF-κB signaling, inflammatory cascades	Increased proliferation, reduced apoptosis, DNA damage	(36)
Oxidative Stress	Excess ROS production by pathogens and inflammatory cells	DNA, protein, and lipid damage	Mutagenesis and genomic instability	(37)
Barrier Dysfunction (“Leaky Gut”)	Increased intestinal permeability	LPS-TLR4-NF-κB signaling	Chronic inflammation and tumorigenesis	(34)
Secondary Bile Acids	Microbial metabolism of dietary fats	Oxidative stress, DNA damage	Tumor promotion	(35)
TMAO	Microbial metabolite linked to inflammation	Pro-inflammatory signaling pathways	Associated with pro-inflammatory and pro-tumorigenic pathways	(37)
Immune Dysregulation	Altered host-microbiota interactions	TLRs, NOD-like receptors	Defective immune surveillance and tumor-promoting inflammation	(36)
Epigenetic Alterations	Microbial metabolites regulate gene expression	DNA methylation, histone modification, ncRNA regulation	Modulation of tumor suppressor and oncogenic pathways	(38, 39)
Butyrate (SCFA)	Histone deacetylase inhibition	Epigenetic regulation, anti-inflammatory signaling	Tumor suppression	(38)
Tumor Microenvironment Modulation	Microbiota influences stromal and immune cells	Cytokine signaling, angiogenesis, immune infiltration	Tumor progression and metastasis	(40)
Gut-Brain Axis	Microbial metabolites influence neuroimmune signaling	Neural and immune pathways	Modulation of inflammation and tumor progression	(40)

Comparing Mediterranean and Western dietary patterns yields important insights into diet-microbiota interactions. The Mediterranean diet is rich in fruits, vegetables, legumes, whole grains, olive oil, and fish, with processed foods and red meat consumed in moderation. This dietary pattern has been associated with favorable gut microbial composition and anti-inflammatory effects (30). On the other hand, Western dietary patterns cause dysbiosis and increased intestinal permeability and promote the production of potentially carcinogenic metabolites. These results suggest that dietary intervention to restore microbial balance may be a viable strategy for CRC prevention.

Ultra-processed foods (UPFs) are becoming more prevalent in our modern diet and have recently been scrutinised. UPFs usually contain high levels of additives, emulsifiers, refined sugars and unhealthy fats and are low in dietary fibre. There is now emerging evidence that UPF is deleterious to gut microbial composition by decreasing beneficial species abundance like Akkermansia muciniphila and Faecalibacterium prausnitzii and increasing pro-inflammatory microorganisms (31). These changes result in chronic low-grade inflammation, impaired barrier function, and increased intestinal permeability that might promote colorectal carcinogenesis. Furthermore, food additives and emulsifiers commonly present in UPFs have been shown to impair mucosal barrier function and modulate host-microbiota interactions, providing additional mechanistic links between modern dietary habits and CRC risk (31).

The metabolism of dietary proteins and fats by microbial flora, in addition to fibre and SCFAs, is also important in colorectal carcinogenesis. High consumption of animal protein and saturated fat enhances the supply of substrates that can be converted into potentially harmful metabolites by gut bacteria. Protein fermentation yields compounds such as ammonia, hydrogen sulphide, phenols and amines, many of which are pro-inflammatory and genotoxic (26). In the same way, dietary fat stimulates the secretion of bile acids and so enhances the production of secondary bile acids by microbial metabolism. Secondary bile acids have been linked to DNA damage, oxidative stress and promotion of tumorigenesis in the colon (26).

Metabolic reprogramming is another important link between nutrition and the development of colorectal cancer. CRC cells undergo extensive metabolic reprogramming that support rapid proliferation and survival in nutrient-deprived settings. There is growing evidence that dietary factors influence the metabolic behaviour of intestinal stem cells, which are considered to be the main cells of origin for CRC (32). The interactions of dietary nutrients, microbial metabolites, immune cells and stromal components create a complex metabolic ecosystem that modulates tumour initiation and progression. Understanding these interactions may offer opportunities for targeted nutritional interventions and metabolic therapies.

Prebiotics are a potential strategy to modulate microbial metabolism and decrease CRC risk. Prebiotics enhance the proliferation of beneficial microorganisms, particularly bifidobacteria and other SCFA-producing taxa (33). Several experimental studies have shown that the administration of specific prebiotic compounds reduces tumour incidence with often beneficial changes in microbiota composition and metabolite production (33). These findings are in support of the concept that dietary modification of microbial communities may play a role in the prevention of colorectal cancer.

In summary, the present evidence strongly supports a central role for diet-driven microbial metabolism in colorectal carcinogenesis. Dietary fibre, SCFA production, beneficial microbial taxa, and Mediterranean dietary patterns appear to be protective against CRC. In contrast, Western diets, ultra-processed foods, high meat intake, and dysbiosis-associated metabolites are associated with carcinogenesis. These observations underscore the importance of viewing the diet-microbiota-metabolite axis as a dynamic and modifiable determinant of colorectal cancer risk. Future prevention strategies should focus on dietary interventions to increase microbial diversity, SCFA production and decrease the formation of pro-inflammatory and carcinogenic metabolites to promote long-term colorectal health (**Table 3**).

**Table 3:** Microbial target/intervention, mechanisms and potential clinical utility.

Application	Microbial target/intervention	Mechanism	Potential clinical utility	Sources
Diagnostic biomarkers	*Fusobacterium nucleatum*, ETBF, pks+ *E. coli*, *Peptostreptococcus anaerobius*	Enrichment in CRC-associated microbiota	Non-invasive CRC detection and risk stratification	(34, 35)
Protective microbial biomarkers	*Faecalibacterium prausnitzii*, *Bifidobacterium* spp., *Lactobacillus* spp.	Reduced abundance in CRC patients	Indicators of intestinal health and lower CRC risk	(37)
Metabolite biomarkers	SCFAs (butyrate, propionate, acetate)	Reflect microbial metabolic activity and gut homeostasis	Biomarkers of dysbiosis and CRC risk	(28)
Harmful metabolite biomarkers	Secondary bile acids	DNA damage, oxidative stress, tumor promotion	Potential prognostic and diagnostic biomarkers	(35)
Prognostic biomarkers	*Fusobacterium nucleatum*	Associated with advanced disease and poorer survival	Prognostic risk stratification	(34)
Multi-omics biomarker platforms	Metagenomics, transcriptomics, proteomics, metabolomics	Comprehensive microbial profiling	Improved diagnostic accuracy and precision medicine	(35)
Traditional probiotics	*Lactobacillus*, *Bifidobacterium*	Restore diversity, improve barrier integrity, modulate immunity	CRC prevention and adjunctive therapy	(41, 42)
*Lactobacillus*spp.	Barrier protection, anti-inflammatory effects, pathogen inhibition	Enhances intestinal homeostasis	Potential adjunctive CRC therapy	(42)
*Bifidobacterium*spp.	SCFA production and immune regulation	Anti-inflammatory activity	CRC prevention and microbiome restoration	(42)
Next-generation probiotics	*Lactobacillus intestinalis*	Activates CCL5 signaling, recruits dendritic cells, enhances antitumor immunity	Targeted microbial immunotherapy	(43)
Targeted microbial therapy	*Bifidobacterium breve*	Produces indole-3-lactic acid (ILA), activates AhR signaling, regulates macrophages	Prevention of inflammation-associated CRC	(44)
Postbiotics	SCFAs, indoles, bacteriocins, extracellular vesicles	Direct modulation of immune and signaling pathways	Stable microbiome-derived therapeutics	(41)
Prebiotics	Dietary fiber and fermentable substrates	Increase bifidobacteria and SCFA-producing bacteria	Prevention of dysbiosis and CRC	(33)
Fecal microbiota transplantation (FMT)	Whole microbial ecosystem transfer	Restores microbial diversity and function	Emerging therapy for CRC and IBD	(35, 45)
Metabolic pathway targeting	Microbial bile acid and tryptophan metabolism	Modulation of carcinogenic pathways	Precision microbiome therapy	(46)
Akkermansia-mediated therapy	*Akkermansia muciniphila*enrichment by Ginsenoside Rh4	Increased UDCA production, FXR activation, reduced inflammation	Novel metabolic intervention	(46)
Precision microbiome medicine	Individual microbiome profiling	Tailored preventive and therapeutic strategies	Personalized CRC management	(29)

## Gut microbiota, chronic inflammation and colorectal carcinogenesis: new mechanisms and progress

Healthy intestinal microbiota is an important modulator of mucosal homeostasis. Microorganisms in the gut participate in barrier integrity, nutrient metabolism, and immune surveillance through interactions with epithelial cells, immune cells, and dietary substrates. Physiologic levels of microbial communities promote tolerance and maintain a balanced inflammatory state. But, this balance can be disturbed, resulting in dysbiosis, which is characterised by a decrease in microbial diversity, loss of beneficial microbes, and proliferation of potentially pathogenic taxa. Such changes lead to a pro-inflammatory microenvironment that promotes colorectal carcinogenesis (34, 35).

The most common conceptual framework describing the association between dysbiosis and CRC is the “driver-passenger” model. This hypothesis states that certain pathogenic microorganisms are the first “drivers” of DNA damage, inflammation and epithelial dysfunction that enable tumour initiation. During tumour progression, the altered microenvironment facilitates the growth of secondary “passenger” bacteria, which further promote cancer progression (34). This model explains the common finding of different microbial communities in different stages of colorectal tumorigenesis.

CRC pathogenesis has been consistently linked to several bacterial species. One of the most studied is the anaerobic bacterium *Fusobacterium nucleatum*, which is often enriched in colorectal tumours. *F. nucleatum* facilitates carcinogenesis through multiple mechanisms including activation of inflammatory signalling pathways, stimulation of cellular proliferation, induction of immune suppression, and facilitation of tumour invasion (34, 36). Experimental studies have shown that *F. nucleatum* induces NF-κB signalling activation and promotes the secretion of pro-inflammatory cytokines including IL-6, IL-8 and TNF-α, thus creating a tumor-promoting microenvironment. Moreover, this microbe can suppress cytotoxic T-cell activity and promote immune evasion in the tumour microenvironment to inhibit antitumor immunity (37).

Another important pathogen associated with CRC is enterotoxigenic Bacteroides fragilis (ETBF). ETBF produces a toxin called Bacteroides fragilis toxin (BFT) that disrupts epithelial barrier integrity and activates inflammatory signalling pathways. BFT exposure induced production of IL-17 and activated STAT3 signalling, which promoted chronic inflammation and contributed to colorectal carcinogenesis (34). Inflammation induced by ETBF can accelerate tumour development and progression from precancerous lesions to invasive malignancy, as demonstrated in experimental models.

Some strains of Escherichia coli are also implicated in CRC development. pks-positive (pks+) Escherichia coli strains are of particular interest because they produce the genotoxin colibactin. Colibactin directly damages host DNA by causing double-strand breaks and mutational signatures that have been identified in human colorectal cancers (36, 37). This is strong evidence that products of microbes can directly influence genomic instability, a hallmark of cancer development. In addition to genotoxic effects, pathogenic E. strains promote inflammation and oxidative stress, further enhancing carcinogenic processes.

Many of the CRC-associated microorganisms act through chronic inflammation as the common pathway. Chronic activation of inflammatory signalling pathways results in increased production of cytokines, chemokines and reactive oxygen species (ROS). Whereas acute inflammation is protective, chronic inflammation induces cellular proliferation, inhibits apoptosis and increases DNA damage. These processes establish a microenvironment that favours malignant transformation (36). The inflammatory tumour microenvironment also recruits immune cells that may paradoxically be supportive of tumour growth through secretion of growth factors and immunosuppressive mediators.

Oxidative stress is another important mechanism that connects dysbiosis and carcinogenesis. Many pathogenic microorganisms directly or by activation of inflammatory pathways induce production of ROS. Excessive ROS can damage DNA, proteins and lipids, resulting in mutations and genomic instability. Accumulation of oxidative damage over time gives rise to the adenoma-carcinoma sequence characteristic of CRC progression (37). Furthermore, oxidative stress can affect epigenetic regulation and change gene expression patterns related to tumour development.

The intestinal barrier is crucial in maintaining the separation of luminal microorganisms from host tissues. This loss of epithelial barrier integrity is often associated with dysbiosis that causes increased intestinal permeability. This phenomenon, commonly referred to as “leaky gut,” allows bacterial products, such as lipopolysaccharide (LPS), to translocate into underlying tissues and systemic circulation. LPS activates Toll-like receptor 4 (TLR4) signalling, resulting in the activation of NF-κB and chronic inflammation (34). Their continued activation promotes epithelial proliferation and tumorigenesis.

Microbial metabolites also have profound effects on colorectal carcinogenesis. Good metabolites such as butyrate have anti-inflammatory and antitumor effects, but other microbial products promote cancer development. Bacterial metabolism of dietary fats into secondary bile acids has been linked to DNA damage, oxidative stress and promotion of tumour growth (35). Similarly, metabolites such as trimethylamine-N-oxide (TMAO) have been linked to inflammation and carcinogenic signalling pathways (37). Thus, alterations in microbial metabolic activity may have a profound impact on cancer risk independent of alterations in microbial composition.

The crosstalk between the microbiota and the immune system is particularly relevant in CRC. The intestinal mucosa harbours a complex network of innate and adaptive immune cells that constantly communicate with microbial signals. Pattern recognition receptors such as Toll-like receptors and NOD-like receptors recognise microbial components and modulate immune responses. Dysbiosis influences these signalling pathways resulting in chronic activation of inflammatory cascades and defective immune surveillance (36). Tumor-promoting inflammation is frequently associated with the suppression of effective anti-tumor immunity, creating a milieu that is conducive to cancer progression.

Recent evidence suggests that epigenetic mechanisms may be an important interface between the microbiota and host carcinogenesis. Gut microorganisms and their metabolites can affect DNA methylation, histone modification and non-coding RNA expression, thus changing gene transcription without changing DNA sequence (38). These epigenetic modifications impact pathways involved in inflammation, immune regulation, cellular proliferation and apoptosis. Short-chain fatty acids (SCFAs), especially butyrate, are histone deacetylase inhibitors and able to modulate the expression of tumor-suppressor genes. Alternatively, metabolites associated with dysbiosis might drive epigenetic alterations that support tumorigenesis (38, 39).

The tumour microenvironment itself is increasingly appreciated as an ecosystem influenced by microbial forces. During disease progression, cancer‐associated fibroblasts, immune cells, endothelial cells and microbial communities dynamically interact. The gut microbiota can influence cytokine production, angiogenesis, immune cell infiltration and metastatic potential (40). Recent studies have also explored the role of neuroimmune pathways and the gut-brain axis in CRC, suggesting that microbial metabolites and neuroimmune signalling may influence inflammation, immune responses and tumour progression (40).

**Figure 1:**
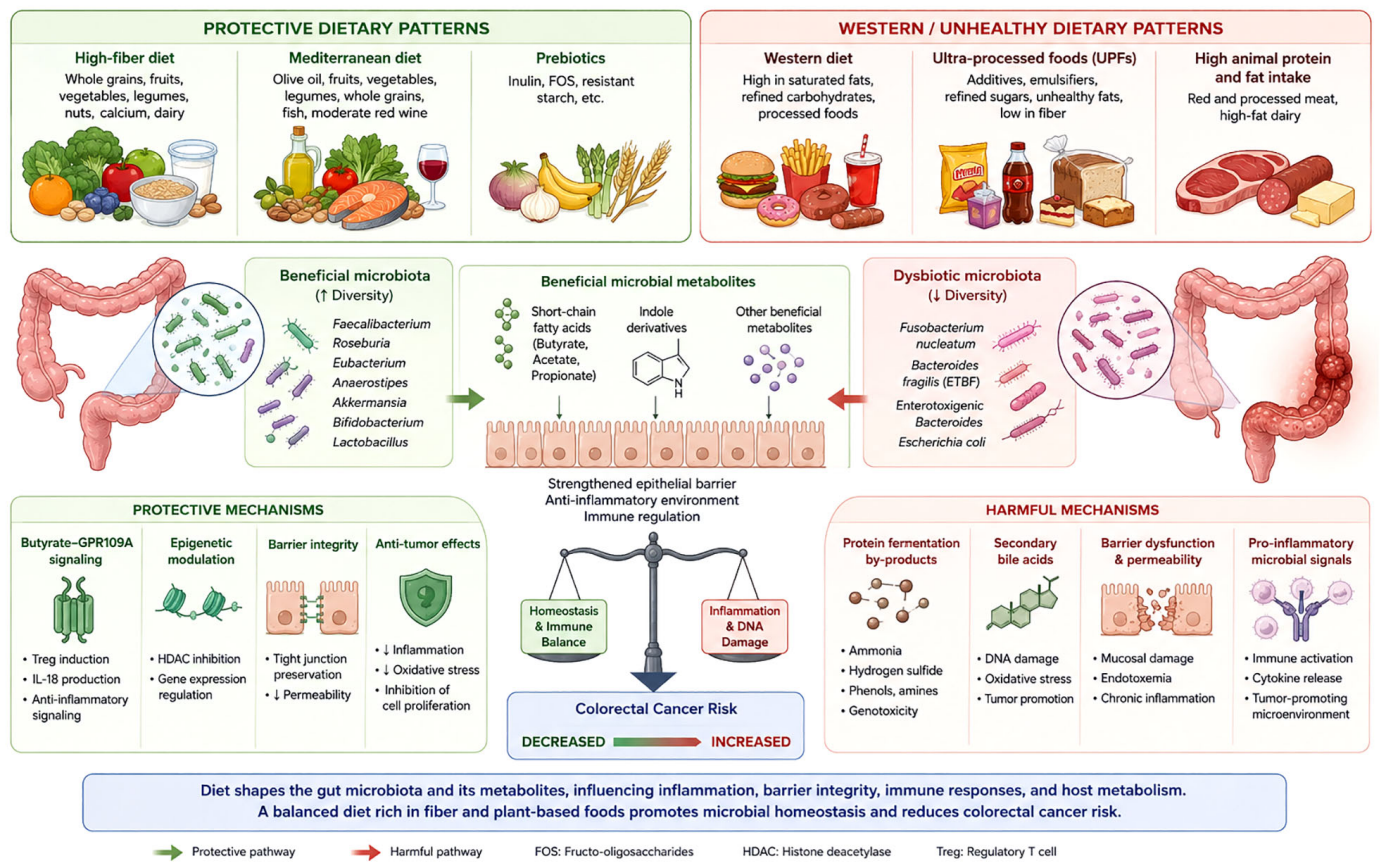
Dietary factors, gut microbiota and metabolic pathways in colorectal cancer risk.

**Figure 2:**
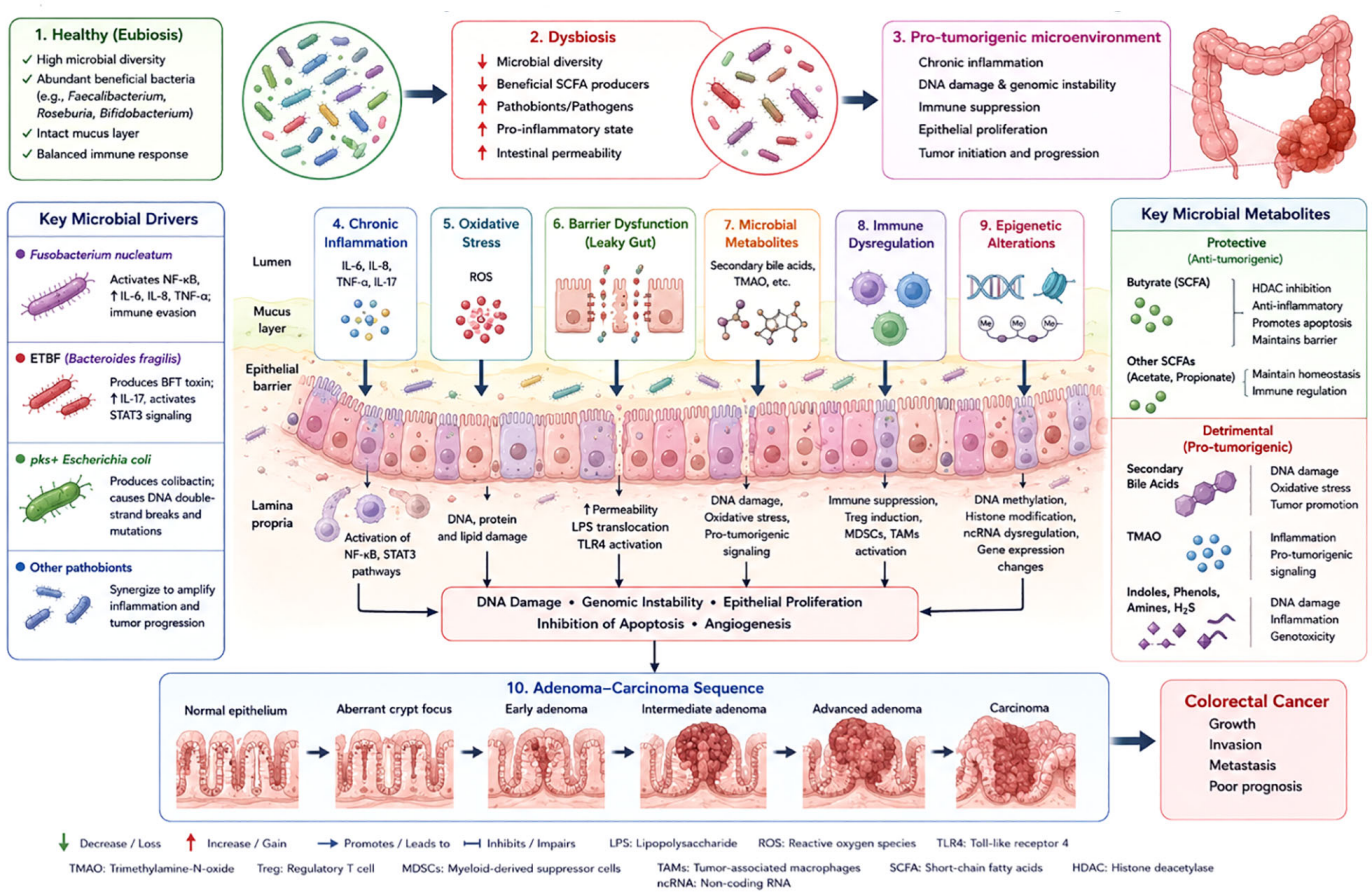
Gut microbiota dysbiosis and mechanisms of colorectal carcinogenesis.

In conclusion, the current evidence suggests that chronic inflammation is the major link between dysbiosis and colorectal carcinogenesis. Certain pathogens, including *Fusobacterium nucleatum*, enterotoxigenic *Bacteroides fragilis* and pks-positive *Escherichia coli*, contribute to tumour initiation through inflammatory, genotoxic and immune-modulatory mechanisms. At the same time, impairment of barrier function, oxidative stress, altered microbial metabolism and epigenetic dysregulation also promote malignant transformation. These findings provide evidence for the notion that CRC is a disease of disrupted host-microbiota interactions in addition to being a genetic disease. Further exploration of these mechanisms will be critical for the identification of novel biomarkers and microbiome-targeted interventions for the prevention or reversal of colorectal carcinogenesis (**Figures 1** and **2**).

## Translational research, biomarkers and microbiome based interventions

The increasing awareness of the gut microbiota’s role in the pathogenesis of CRC has turned microbiome research from a purely descriptive discipline into a fast-evolving field of translational medicine. We have made significant advances in our understanding of the role of microbial dysbiosis in colorectal carcinogenesis, and current efforts are increasingly focused on translating these findings into clinical utility. Such applications include microbiome-based biomarkers for early diagnosis and prognosis, therapeutic manipulation of microbial communities, and personalised interventions to restore intestinal homeostasis. Advances in sequencing technology, metabolomics, and systems biology have accelerated the identification of microbial signatures associated with CRC, opening avenues for precision medicine approaches that complement traditional diagnostic and therapeutic strategies.

One of the most promising translational applications of microbiome research is the development of microbial biomarkers for CRC detection. Screening methods including colonoscopy and faecal occult blood testing remain important tools for CRC prevention and diagnosis. However, these approaches are limited by invasiveness, cost, patient compliance and diagnostic sensitivity. Emerging evidence indicates that specific changes in the composition of gut microbiota might serve as non-invasive biomarkers for identifying individuals at increased risk for CRC or detecting early stage disease (35).

Several studies have reported that some bacterial taxa are consistently enriched in CRC patients. Some species such as *Fusobacterium nucleatum*, enterotoxigenic *Bacteroides fragilis*, pks-positive *Escherichia coli* and *Peptostreptococcus anaerobius* are often found in association with colorectal tumours and precancerous lesions (34, 35). Conversely, CRC patients have decreased levels of beneficial microbes such as *Faecalibacterium prausnitzii*, *Bifidobacterium* species, and *Lactobacillus* species (37). Such reproducible changes in microbes provide the basis for development of microbiome-based diagnostic algorithms to distinguish healthy individuals from those with adenomas or cancer.

Besides microbial composition, microbial metabolites are emerging as highly informative biomarkers. Butyrate, propionate and acetate (short-chain fatty acids, SCFAs) are end products of the metabolism of beneficial fermentative bacteria and are important for the maintenance of intestinal homeostasis (28). Reduced concentrations of SCFA have been consistently associated with dysbiosis, inflammation and colorectal carcinogenesis Therefore, SCFA levels could be a useful indicator of both microbial function and disease risk. Similarly, increased levels of secondary bile acids and other carcinogenic metabolites have been associated with CRC progression, indicating that metabolomic profiling may be a complementary approach to microbial sequencing approaches in the future diagnostic strategies (35).

There is also a lot of interest in the idea of microbiome-based prognostic biomarkers. Disease stage, tumour aggressiveness, treatment response, and survival outcomes are linked to specific microbial signatures. For example, an increased abundance of *Fusobacterium nucleatum* has been associated with advanced disease and poorer prognosis in several studies (34). Stratifying patients according to their microbiome features could potentially lead to the selection of personalised therapies and more precise risk prediction in the future.

Next generation sequencing technologies have greatly accelerated the discovery of biomarkers. High-throughput metagenomic sequencing is used to characterise microbial communities in its entirety. Transcriptomic, proteomic, and metabolomic approaches are used to gain insight into microbial functionality. These technologies allow the detection of complex microbial signatures, instead of relying on individual bacterial species. These multi-dimensional biomarkers may have better diagnostic performance than traditional single marker approaches (35).

In parallel to the development of biomarkers, considerable effort has been made to therapeutically modulate the gut microbiota. Probiotics are one of the most studied interventions and are defined as live microorganisms that, when administered in adequate amounts, confer a health benefit on the host. Probiotics may affect CRC risk through various mechanisms, including the restoration of microbial diversity, improvement of barrier function, immune response modulation, and production of beneficial metabolites (41).

Traditional probiotic genera, such as *Lactobacillus* and *Bifidobacterium*, have shown significant potential in both experimental and clinical studies. Lactobacillus species contribute to the integrity of epithelial barrier, inhibit inflammatory signalling pathways, inhibit pathogen colonisation and modulate the host immune response (42). Thus, Bifidobacterium species are involved in SCFA production and anti-inflammatory action, contributing to intestinal homeostasis. These properties render them attractive candidates for CRC prevention and adjunctive therapy.

Recent research has moved beyond traditional probiotics to next-generation probiotics and to targeted microbial therapies. A well-known example is Lactobacillus intestinalis, which has been shown to suppress tumour growth in both chemically induced and genetically driven CRC models (43). Mechanistically, L. intestinalis promotes dendritic-cell infiltration in the tumour microenvironment through activation of the CCL5 signalling pathway, thereby enhancing antitumor immune responses. These results suggest that specific microbial strains can directly affect tumour immunity and have the potential to enhance cancer therapy.

Bifidobacterium breve has also shown significant antitumor effects in models of colitis-associated cancer (44). Investigators found that Bifidobacterium breve stimulates the production of indole-3-lactic acid (ILA), a metabolite of tryptophan metabolism. ILA shifts macrophage differentiation toward anti-inflammatory phenotypes and inhibits tumor-promoting inflammation via activation of the aryl hydrocarbon receptor pathway. This work underscores the importance of microbial metabolites as mediators of therapeutic benefit, and suggests that microbial-derived bioactive compounds themselves may be potential future therapeutic agents.

Another area of emerging interest is the use of postbiotics, which are defined as bioactive microbial products or metabolites that provide health benefits independent of live microorganisms. Postbiotics have some theoretical advantages over traditional probiotics in terms of stability, standardisation and safety. SCFAs, indoles, bacteriocins, and extracellular vesicles are beneficial compounds that can directly modulate host immune responses and cellular signalling pathways involved in carcinogenesis (41). With the progress in the knowledge of microbial metabolite biology, postbiotics may become an important component of microbiome-based therapeutic strategies.

Another promising intervention is the use of prebiotics. Prebiotics, in contrast to probiotics, selectively stimulate the growth and activity of beneficial endogenous microorganisms. Dietary fibres and other fermentable substrates increase the population of bifidobacteria and SCFA-producing bacteria, resulting in normobiosis and reduced inflammation (33). Experimental studies have shown reduced tumour incidence and favourable alterations in microbial composition with the administration of some prebiotic compounds, thus supporting their role in CRC prevention.

One of the most powerful ways to manipulate the gut microbiome has become faecal microbiota transplantation (FMT). FMT can rapidly restore microbial diversity and function by transferring an entire microbial ecosystem from a healthy donor to a recipient. Although predominantly established for recurrent Clostridioides difficile infection at present, FMT is increasingly studied in CRC and inflammatory bowel disease (35, 45). Animal studies suggest that FMT can restore dysbiosis, reduce inflammation and alter responses to anti-cancer therapies. But issues of donor selection, safety, standardisation and long-term effects need to be worked out before it can be widely applied in clinical practice.

Microbiota-mediated metabolic interventions are a particularly exciting area of translational research. Recent studies have shown specific microbial communities regulate bile acid metabolism, production of short-chain fatty acids (SCFAs), tryptophan metabolism and other pathways with direct effects on carcinogenesis. For example, Ginsenoside Rh4 suppresses CRC by enriching Akkermansia muciniphila and promoting the production of ursodeoxycholic acid (UDCA) to activate FXR signalling and inhibit inflammatory pathways (46). Such findings suggest that targeting microbial metabolic pathways may be a particularly specific therapeutic strategy.

The future of precision medicine is the future of microbiome-based oncology. Emerging approaches, instead of universal interventions, seek to characterise individual microbiome profiles and tailor therapies accordingly. Baseline differences in microbial composition affect responses to probiotics, dietary interventions, immunotherapy, and other treatments (29). Therefore, the combination of microbiome profiling with genomic, transcriptomic and clinical data could allow for highly personalised prevention and treatment strategies.

While progress is being made, there are a lot of challenges. Microbiome composition is highly variable across populations, geographic regions, dietary habits and individuals. For microbiome biomarkers to be implemented routinely in clinical practice, standardisation of sampling methods, sequencing technologies, analytical pipelines and reporting standards is needed. Furthermore, mechanistic studies and large-scale clinical trials are needed to further explore the causal relationships between microbial alterations and disease progression.

In summary, translational microbiome research is rapidly changing the landscape of prevention, diagnosis and treatment of colorectal cancer. Microbial signatures and metabolites hold promise as non-invasive biomarkers for CRC detection and prognosis. At the same time, probiotics, prebiotics, postbiotics, faecal microbiota transplantation and targeted microbial therapies provide new avenues to modulate carcinogenic pathways and restore intestinal homeostasis. With ongoing technological improvements increasing our understanding of host-microbiota interactions, microbiome-based precision medicine is ready to be part of the future of colorectal cancer management strategies.

## Precision nutrition

Precision nutrition is an emerging area of microbiome-guided medicine in which the aim is to tailor dietary interventions to an individual’s genetics, metabolic profile, tumour biology and composition of the gut microbiome. Precision nutrition uses genomic, transcriptomic, metabolomic and microbiome data to define personalised nutritional strategies that may reduce colorectal cancer risk or improve therapeutic outcomes, rather than relying on broad dietary guidelines (47). Recent evidence has shown that changes in lipid metabolism are very promising therapeutic targets as dietary components can modulate metabolic pathways that are involved in tumour growth, progression and metastasis (47). The use of precision nutrition to reduce recurrence risk through nutrient-gene interactions and management of metabolic heterogeneity across tumours is also being explored and is supportive of precision oncology strategies (48). In support of this, bioactive compounds from food, especially saponin-rich extracts, have been shown to have the ability to selectively modulate colorectal cancer metabolism, inhibit lipid metabolic pathways, and enhance the efficacy of standard chemotherapy in preclinical models, highlighting the potential of personalised dietary interventions as adjunctive therapeutic strategies (49). Despite these promising advances, most of the evidence remains preclinical or mechanistic in nature, and prospective clinical trials are required before the application of precision nutrition in routine prevention or treatment of colorectal cancer (47–49).

## Gut microbiome-based biomarkers

Gut microbiome-based biomarkers are emerging as promising non-invasive tools for the early detection of CRC, and several studies have demonstrated that specific microbial signatures can differentiate CRC patients or patients with advanced adenomas from healthy controls (50, 51). Microbiome-based models, especially faecal microbiome-based models, have shown promising diagnostic performance, and may enhance screening accuracy combined with conventional tests such as the faecal occult blood test or faecal immunochemical test (50). Yet, even with these advances, there remain substantial barriers to be cleared before microbiome-derived biomarkers can be integrated into routine clinical practice. Reproducibility and external validation across studies has been hampered by high variability in sampling techniques, sequencing technologies, bioinformatic workflows, study cohorts and microbial differences among individuals (50, 51). Therefore, although gut microbiota signatures constitute a promising addition to current screening strategies for colorectal cancer, large prospective multicentre studies and standardised analytical protocols are needed to evaluate their clinical utility (50, 51).

## Artificial intelligence and gut microbiome

Artificial intelligence is becoming a powerful tool to interrogate the complexities of the gut microbiome, and to help the development of personalised diagnostics and therapeutic strategies. By integrating large microbiome and multi-omics datasets with machine learning and deep learning algorithms, microbial signatures can be identified that are associated with disease risk, as well as treatment response prediction and discovery of novel live biotherapeutic products (54). Besides the discovery of biomarkers, the application of AI in microbiome analysis has shown promising clinical applications such as the production of personalised dietary interventions based on the microbiome profile of individuals. AI-guided microbiome-based diets have been shown to improve gastrointestinal symptoms, quality of life and microbiome composition in randomised controlled trials, indicating that precision dietary modulation is more effective than traditional management strategies (52, 55). At the same time, AI-enabled technologies such as automated Raman-activated cell sorting enable high-throughput screening and isolation of functionally relevant microbes at the single-cell level, opening up new possibilities for microbiome characterisation and therapeutic development (53). However, before routine integration of AI-assisted microbiome analysis into precision oncology, issues related to data standardisation, model interpretability, external validation and integration into clinical workflows need to be addressed (52–55).

## Discussion

This review presents the increasing evidence on the interrelated roles of diet, gut microbiota, microbial metabolism and chronic inflammation in colorectal carcinogenesis. Emerging data indicate that dietary patterns affect the composition and function of the gut microbiota, resulting in changes in microbial metabolites that may promote or inhibit carcinogenesis. High fibre dietary patterns and the generation of short-chain fatty acids (SCFA) appear protective, while Western dietary patterns, ultra-processed foods and metabolites associated with dysbiosis have been associated with a pro-inflammatory and potentially carcinogenic intestinal environment (25, 26, 30, 31). In addition, there is increasing mechanistic evidence that microbial dysbiosis promotes colorectal carcinogenesis through inflammatory, genotoxic, metabolic and immune-mediated pathways involving organisms such as *Fusobacterium nucleatum*, enterotoxigenic *Bacteroides fragilis* and pks-positive *Escherichia coli* (34, 36, 37).

A major take-away from this review is that CRC can no longer be considered as a disease driven solely by host genetic alterations. Rather, it appears to develop from a complex interaction of host genetics, environmental exposure, dietary factors, microbial communities and immune responses. The classical adenoma-carcinoma sequence remains the basis of the understanding of colorectal carcinogenesis, but emerging evidence indicates that microbial factors may influence several steps in this sequence by modulating epithelial barrier function, inflammatory signalling pathways, oxidative stress, epigenetic regulation and tumour microenvironment dynamics (38–40). This wider view offers possibilities for preventive and therapeutic measures that go beyond traditional oncologic approaches.

Many important uncertainties remain, despite the huge progress in microbiome research. First, most of the human evidence is observational, so it is difficult to make definitive statements about causality. The role of microbial dysbiosis in the initiation of colorectal carcinogenesis as a consequence of tumour progression or both is not completely understood. Second, there is considerable methodological heterogeneity across studies, including differences in stool versus tissue sampling, sequencing technologies, bioinformatic pipelines, and taxonomic classification, which hampers direct comparison and limits reproducibility. There are also many confounding factors affecting the composition of the microbiome such as diet, antibiotic exposure, age, ethnicity, geography and comorbidities which are variably controlled for in different studies. Thus, while mechanistic studies provide a great deal of support for biological plausibility, translation into clinically relevant biomarkers and therapeutic strategies requires much more rigorous prospective evidence.

The review also supports the notion that microbial metabolites are an important mechanistic interface between diet and colorectal carcinogenesis. Among these metabolites, butyrate is one of the most studied ones due to its anti-inflammatory, immunomodulatory, and epigenetic effects (27, 28). Yet, it is becoming increasingly clear that the metabolic consequences of dysbiosis are far from limited to SCFA depletion. Secondary bile acids, trimethylamine-N-oxide (TMAO), hydrogen sulphide and other microbially-derived compounds may play a role in genomic instability, chronic inflammation and tumour progression (26, 35, 37). These results highlight that microbial functionality may be as important as microbial composition itself and emphasise the need for future studies to integrate metabolomic and metagenomic approaches, rather than focusing exclusively on taxonomic changes.

The driver–passenger model is a useful conceptual framework but does not provide evidence for a causal relationship between microbial dysbiosis and colorectal carcinogenesis. Most of the human data available are from cross-sectional and observational studies, and it is difficult to determine whether microbial changes are the cause of tumour development or are a consequence of the evolving tumour microenvironment. Despite persuasive mechanistic evidence (56), longitudinal human studies are scarce and the causal link between dysbiosis and colorectal cancer remains controversial. Moreover, recent evidence suggests that the physicochemical properties of dietary substrates, rather than their mere availability, play a significant role in microbial metabolism. Dietary polysaccharides can vary in structure and molecular weight, which can have differential effects on microbial fermentation rates, community composition and production of bioactive metabolites. This is an example of the complexity of diet–microbiota interactions beyond short-chain fatty acid production. These observations support the concept that dietary modulation of microbial metabolic networks may be a major target for future CRC prevention strategies (57). Also, The growing body of evidence indicates that the gut microbiota, microbial metabolites, and host immune responses interact with each other to dynamically modulate the integrity of the intestinal barrier. Beneficial microbial communities stimulate mucus secretion, maintain tight junction integrity, and reduce intestinal inflammation, while dysbiosis impairs these protective mechanisms, increases epithelial permeability, and allows for microbial translocation. This in turn promotes the innate and adaptive immune activation leading to a vicious cycle of barrier dysfunction and chronic inflammation promoting colorectal carcinogenesis. Experimental studies indicate that re-establishing favourable microbial communities may restore mucosal barrier function and decrease inflammatory and oxidative stress responses, making the gut microbiota and mucosal barrier axis a potential therapeutic target (58).

Recent experimental evidence supports the reciprocal relationship between gut microbiota and intestinal barrier integrity. It has been shown that regulation of gut microbiota is effective to reduce intestinal inflammation and oxidative stress, increase the expression of tight junction proteins (Claudin-1, Occludin and ZO-1) and limit lipopolysaccharides translocation through the intestinal epithelium. These alterations maintain the function of the mucosal barrier and decrease immune activation, highlighting the concept that the maintenance of intestinal barrier integrity is an important mechanism underlying the alleviation of chronic inflammation and colorectal carcinogenesis by microbiota-targeted interventions (59).

A major translational implication of the current literature is the growing potential of microbiome-based biomarkers. Several microbial signatures were associated with CRC development, disease stage and prognosis, indicating the potential of microbiome profiling for future screening and risk stratification strategies (34, 35). Similarly, the development of sequencing technologies and multi-omics platforms has facilitated the identification of microbial patterns that could enhance diagnostic performance beyond traditional single-marker approaches. Despite this promise, most candidate biomarkers are still investigational and need external validation before they can be routinely implemented in the clinic.

Another fast-growing area is therapeutic manipulation of the gut microbiota. Preclinical and early clinical data for traditional probiotics, prebiotics, postbiotics, faecal microbiota transplantation and next generation microbial therapies have been promising (33, 41, 43, 44). However, majority of evidence supporting these interventions comes from experimental models, animal experiments or small clinical cohorts. Thus, although microbiome-targeted therapies are attractive, their long-term efficacy, optimal patient selection, safety profile and reproducibility are not fully understood. These approaches still require large randomised clinical trials before they can be incorporated into standard algorithms for CRC prevention or treatment.

Collectively, the evidence reviewed points to dietary modulation of the gut microbiota as one of the most feasible and immediately applicable approaches for prevention of colorectal cancer. Although the development of microbiome-based biomarkers, next generation probiotics, postbiotics and targeted microbial therapies has shown promising results, most of them are still in the investigational stage and need more validation before being used clinically. In contrast, dietary interventions are already widely available and can directly affect microbial composition, metabolic activity and inflammatory pathways involved in colorectal carcinogenesis. This distinction underscores the necessity of integrating preventive nutrition strategies with innovative microbiome-based technologies. Ultimately, the greatest clinical benefit may come from integrating dietary optimisation with microbiome profiling and targeted interventions to enable a more personalised approach to CRC prevention and management.

## Strengths and limitations

An important feature that distinguishes the present review, and a notable strength, is the integrated analysis of the diet-microbiota-metabolite-inflammation axis within a single translational setting. Previous reviews have often focused on individual components, such as dietary risk factors, microbial dysbiosis, inflammatory mechanisms or microbiome-based therapies in isolation. In contrast, this review integrates evidence from these interrelated fields and highlights effects of dietary exposures on microbial communities, effects of microbial metabolism on inflammatory and carcinogenic pathways, and the application of these mechanistic insights to biomarkers and therapeutic strategies. This review offers a comprehensive perspective that ties together nutritional factors, microbial ecology, chronic inflammation, and novel translational applications that may help in the creation of precision prevention and customised management strategies for colorectal cancer. Despite the significant progress made in the field, there are several limitations in the existing literature that must be recognised. First, studies show significant heterogeneity in microbiome sampling techniques, sequencing platforms, analytical pipelines and bioinformatic methods, rendering direct comparisons difficult. Second, a large number of studies are cross-sectional and observational, which limits the ability to infer causality between microbial alterations and CRC carcinogenesis. Third, microbiome composition is influenced by numerous confounders including age, geography, ethnicity, dietary habits, medication exposures, and lifestyle characteristics, which may partly explain discrepancies among studies. Finally, many mechanistic insights derive from animal models, and their relevance to human disease is uncertain. This review has limitations as well. Being a narrative review, it is by nature subject to selection bias and does not possess the systematic methodology, quantitative synthesis and formal risk-of-bias assessment that are inherent in systematic reviews and meta-analyses. In addition, microbiome research is evolving rapidly and new evidence may change current understanding of the role of specific microbial species, metabolites or therapeutic interventions. However, the narrative approach enabled a broad integration of mechanistic, translational and clinical evidence from diverse disciplines, providing a general overview of a very complex and rapidly changing field.

Microbiota-based interventions have been promising in preclinical studies, but translating them into routine clinical practice is not straightforward. Much of the evidence supporting the use of probiotics, postbiotics, targeted microbial therapies and faecal microbiota transplantation is derived from animal models or small preliminary clinical studies with only a handful of adequately powered randomised controlled trials. Moreover, the high variability in the gut microbiome composition between individuals means that the same interventions could result in very different outcomes in different patients. Additional challenges include the lack of standardised microbial formulations, the unknown optimal dose and duration of treatment, the variability in the choice of donor for faecal microbiota transplantation and the limited knowledge of long-term safety. The regulatory framework for live biotherapeutic products is not yet fully developed, limiting their clinical application.

## Barriers to clinical translation

Despite significant scientific advances, several barriers continue to limit the translation of microbiome research into routine clinical practice. One of the major challenges is the lack of standardization in microbiome sampling methods, sequencing platforms, bioinformatic pipelines, and reporting frameworks, which contributes to variability among studies and complicates comparisons across populations. Geographic variation, cultural differences, dietary habits, medication exposure, and environmental factors can substantially influence microbial composition, limiting the generalizability of findings. Furthermore, marked inter-individual variability in microbiome profiles makes it difficult to define universal microbial biomarkers or therapeutic targets. The high cost and technical complexity of advanced sequencing and multi-omics technologies may also restrict widespread clinical adoption. Finally, regulatory challenges regarding the development, validation, and approval of microbiome-based diagnostics and therapeutics remain largely unresolved. Addressing these barriers will be essential before microbiome-guided approaches can be integrated into routine colorectal cancer prevention, diagnosis, and treatment strategies.

Regulatory and ethical issues, in addition to scientific and technical challenges, must be resolved before widespread implementation of microbiome-targeted therapies. Interventions such as probiotics, live biotherapeutic products and faecal microbiota transplantation need to adhere to strict quality control, standard manufacturing procedures and well-defined regulatory frameworks to ensure safety, efficacy and reproducibility. Ethical issues include informed consent, donor screening and traceability for faecal microbiota transplantation, data privacy concerning microbiome sequencing, and equitable access to personalised microbiome-based therapies. Addressing these issues will be important for the safe integration of microbiome-guided approaches into standard clinical care for colorectal cancer, consistent regulatory standards and ethical oversight.

## Knowledge gaps and future directions

There’s still lots of big controversies that don’t allow progress in this area. It is important to distinguish causal microbial drivers from organisms that preferentially colonise established tumours. The relative importance of microbial composition versus microbial metabolic activity is not yet clear with increasing evidence suggesting that functional changes may give more insight than taxonomical changes alone. Another unresolved issue is the extent to which microbial signatures identified in one population in a specific geographic area can be generalised to other populations given the significant impact of diet, ethnicity and environmental exposures on microbiome composition. Ultimately, there is little consensus on the optimal sampling strategy, sequencing methodology or analytical pipeline, which hampers cross-study reproducibility.

A few avenues for future research are especially worth mentioning. Future studies should focus on longitudinal cohort studies that can explain causal relationships between dietary exposures, microbial changes and CRC development. Standardisation of microbiome sampling, sequencing methodologies and reporting frameworks will be vital to improve reproducibility and enable comparisons between studies. In addition, with the hope of a more complete understanding of host-microbiota interactions, multi-omics approaches including microbiomics, metabolomics, transcriptomics, proteomics, and host genomic data will be available. Special emphasis should also be placed on the identification of microbial signatures predictive of treatment response, immunotherapy efficacy, risk of recurrence and long-term survival. Finally, well-designed randomised clinical trials are required to assess microbiome-targeted interventions, including probiotics, postbiotics, dietary strategies, and faecal microbiota transplantation.

The excitement for therapies targeting the microbiome should be tempered. Preliminary results are encouraging for probiotics, postbiotics, faecal microbiota transplantation and targeted microbial interventions in experimental models and early-phase clinical studies, but evidence from sufficiently powered randomised trials is scarce. The gut microbiome is complex and dynamic, and interventions that work in one population may not be easily transferred to others. Thus, current microbiome-directed therapies should be viewed as experimental strategies with potential rather than established components of colorectal cancer prevention or management.

Innovative multimodal approaches combining gut microbiome profiles with host transcriptomics and histopathological features have demonstrated better prognostic stratification than single-modality approaches. These models can enhance survival predictions and facilitate individualised risk assessment by capturing host–microbe interactions and molecular and tissue-level changes, underscoring the increasing significance of microbiome-informed multi-omics in precision oncology (60).

Future multicentre randomised clinical trials are needed to evaluate microbiome-targeted interventions with standardised protocols, include longitudinal microbiome profiling and determine whether modulation of the gut microbiota can improve clinically meaningful endpoints such as adenoma recurrence, treatment response, disease-free survival and overall survival.

## Ongoing clinical trials

Microbiome-based interventions have shown great promise in preclinical studies but are in the early stages of integration into standard clinical practice. Numerous ongoing clinical trials are assessing the potential for modulation of the gut microbiome to improve clinically meaningful outcomes in patients with CRC. The OPTIMA study (ClinicalTrials.gov identifier: NCT05655780) (61) prospectively investigates the potential of gut microbial ß-glucuronidase activity, in conjunction with tumour molecular subtype and UGT1A1 genotype, to individualise irinotecan-based therapy, predict treatment response and toxicity, and improve survival and quality of life through biomarker-guided treatment strategies. The FiberUP randomised trial (NCT06212817) (62) is testing whether increasing preoperative dietary fibre intake can beneficially modify the gut microbiome and microbial metabolites, while simultaneously decreasing the incidence of postoperative complications in patients undergoing colorectal cancer surgery. Another recent phase I exploratory study (NCT07486492) (63) is investigating faecal microbiota transplantation from young donors with PD-1 inhibition and FOLFIRI chemotherapy in microsatellite-stable metastatic colorectal cancer to determine if microbiome modulation can enhance immunotherapy efficacy in a patient population that normally gains little benefit from immune checkpoint blockade. These studies illustrate the ongoing shift from mechanistic studies to clinically relevant microbiome-guided interventions. Most current trials, however, are focused on feasibility, safety, biomarker validation, or surrogate biological endpoints rather than definitive oncologic outcomes. Adequately powered multi-center randomised trials demonstrating improvements in recurrence, disease-free survival, or overall survival are lacking. Therefore, microbiome-based interventions should be regarded as promising experimental approaches, not as established components of standard colorectal cancer therapy.

## Clinical implications

Although microbiome-targeted interventions are largely investigational, current evidence supports dietary optimisation and preservation of microbial homeostasis as potentially important components of strategies for the prevention of colorectal cancer. Diets rich in fibre, fruits, vegetables and whole grains may contribute to the beneficial microbial populations and metabolic profiles linked to decreased carcinogenic risk. In the future, the integration of microbiome-derived biomarkers with existing screening modalities like faecal immunochemical testing and colonoscopy could improve early detection, risk stratification, and personalised surveillance strategies. Microbiome-informed approaches may complement existing preventive and therapeutic frameworks and guide to more individualised management of colorectal cancer as the evidence evolves. These developments should be considered as an addition to current developments in colorectal cancer management including robotic surgery, molecular profiling and precision oncology. With the evolution of robotic techniques their integration into multidisciplinary, individualised treatment protocols has the potential to improve perioperative outcomes, functional rehabilitation and long-term management of cancer (64–73).

## Conclusion

In conclusion, the diet-microbiota-inflammation axis is likely at the core of colorectal carcinogenesis according to current evidence. Dietary patterns, microbial dysbiosis, microbial metabolites and chronic inflammation are linked in a complex network that affects tumour initiation, progression and clinical outcomes. Progress in microbiome science offers promising avenues for the development of new biomarkers, preventive strategies, and microbiota-targeted therapies, despite major challenges concerning causality, standardisation and clinical implementation. Further integration of mechanistic and translational research is essential to unlock the potential of microbiome-based precision medicine for colorectal cancer. There are still major uncertainties regarding causality, methodological standardisation and clinical validation, highlighting the need for large prospective multicentre studies before microbiome-guided approaches can be routinely implemented.
